# The spliceosome-associated protein Mfap1 binds to VCP in Drosophila

**DOI:** 10.1371/journal.pone.0183733

**Published:** 2017-08-24

**Authors:** Sandra Rode, Henrike Ohm, Jaqueline Zipfel, Sebastian Rumpf

**Affiliations:** Institute for Neurobiology, University of Münster, Badestrasse 9, Münster, Germany; EPFL, SWITZERLAND

## Abstract

Posttranscriptional regulation of gene expression contributes to many developmental transitions. Previously, we found that the AAA chaperone Valosin-Containing Protein (VCP) regulates ecdysone-dependent dendrite pruning of Drosophila class IV dendritic arborization (c4da) neurons via an effect on RNA metabolism. In a search for RNA binding proteins associated with VCP, we identified the spliceosome-associated protein Mfap1, a component of the tri-snRNP complex. Mfap1 is a nucleolar protein in neurons and its levels are regulated by VCP. Mfap1 binds to VCP and TDP-43, a disease-associated RNA-binding protein. via distinct regions in its N- and C-terminal halfs. Similar to *vcp* mutations, Mfap1 overexpression causes c4da neuron dendrite pruning defects and mislocalization of TDP-43 in these cells, but genetic analyses show that Mfap1 is not a crucial VCP target during dendrite pruning. Finally, rescue experiments with a lethal *mfap1* mutant show that the VCP binding region is not essential for Mfap1 function, but may act to increase its stability or activity.

## Introduction

Pruning, the regulated loss of synapses or neurites during neuronal development, is an important specification mechanism that contributes to the mature morphology of neurons [1]. In *Drosophila*, pruning occurs primarily during metamorphosis, when the larval nervous system develops into the adult one. For example, the peripheral sensory class IV dendritic arborization (c4da) neurons prune their extensive larval dendritic arbors during the first sixteen hours after puparium formation (h APF) [2,3]. C4da neuron dendrite pruning is regulated by the steroid hormone ecdysone [2,3]. Ecdysone activates the EcR-B1 isoform of the ecdysone receptor, a transcription factor of the steroid hormone receptor type, leading to the expression of pruning genes such as *Sox14*, *Mical*, and *headcase* [4,5]. These gene expression changes ultimately result in destabilization of dendritic microtubules and the dendritic plasma membrane [6,7] In addition, the ubiquitin-proteasome system (UPS) is also required for dendrite pruning [2]. We previously found that mutations in the UPS chaperone Valosin-Containing Protein (*VCP*, also known as *TER94*), cause defects in dendrite pruning and ecdysone-induced neuronal apoptosis [8,9]. VCP is a AAA ATPase with two consecutive ATPase domains. It recruits ubiquitylated target proteins via a large number of VCP adaptor proteins [10,11]. These target proteins can be unfolded by VCP, usually for degradation within the proteasome [10,11]. During c4da neuron dendrite pruning, VCP inactivation abrogates functional expression of the ecdysone target gene *Mical*, likely through induction of a different Mical splice isoform [8]. In addition, VCP inactivation causes mislocalization of the RNA-binding protein TDP-43 from the cytosol to the nucleus in c4da neurons [8].

Based on these observations, we hypothesized that VCP regulates dendrite pruning via an effect on mRNA metabolism. In a search for splicing factors interacting with VCP, we identified the spliceosome-associated Microfibril-associated protein 1 (Mfap1). Mfap1 binds to Prp38, a component of the tri-small nuclear ribonucleoprotein (tri-snRNP) complex, an intermediate complex during the splicing reaction, and is required for splicing of a cell cycle-related mRNA [12]. In *Caenorhabditis elegans*, mfap-1 mutations cause alterations in alternative splicing of a reporter gene, with both intron retention and exon skipping [13]. The C-terminal half of Mfap1 (amino acids 229–478) contains a conserved motif (Pfam PF06991) that is frequently found in spliceosome components. This region of Mfap1 was shown to be required for Prp38 binding, but no interaction partners have been identified for the N-terminal part [12]. In this study, we found that Mfap1 overexpression inhibits c4da neuron dendrite pruning. Mfap1 levels are increased upon VCP inhibition, and it interacts with VCP and TDP-43 via its N- and C-terminal domains, respectively. Our genetic analysis suggests that Mfap1 is not a VCP target during c4da neuron dendrite pruning. In order to address the relevance of the observed interactions, we generated a lethal *mfap1* mutant. Rescue experiments show that the N-terminal 229 amino acids of Mfap1 containing the VCP binding site are not required for viability, but confer overexpression toxicity in te context of full length Mfap1. Thus, VCP binding may serve to stabilize the spliceosome-associated protein Mfap1.

## Results

### Mfap1 overexpression causes c4da neuron dendrite pruning defects

**Fig 1:**
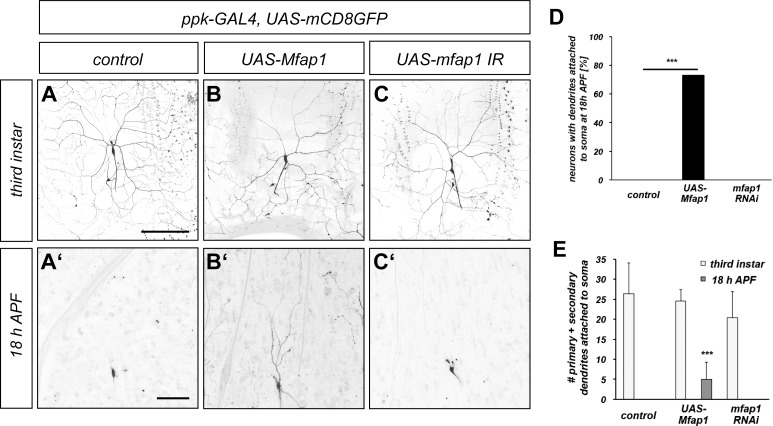
Overexpression of Mfap1 causes c4da neuron dendrite pruning defects. (A)–(C) Mfap1 overexpression causes defects in c4da neuron dendrite pruning. Upper panels (A)–(C) show third instar larval c4da neurons, lower panels (A`)–(C`) show c4da neurons at 18 h APF. C4da neurons were labeled by *ppk-GAL4* driving expression of UAS-mCD8::GFP. (A), (A`) Control c4da neurons. (B), (B`) C4da neurons overexpressing *Mfap1*. (C), (C`) C4da neurons expressing *mfap1* RNAi. (D), (E) Quantification of dendrite pruning defects. (D) Number of neurons with attached dendrites at 18 h APF. *** P<0.0005, Fisher’s exact test. (E) Number of primary and secondary dendrites attached to the soma at third instar (empty bars) or at 18 h APF (full bars). *** P<0.0005, Wilcoxon test. Scale bars are 50 μm.

Based on our previous analysis of the role of VCP during c4da neuron dendrite pruning [8], we hypothesized that VCP might be involved in the inactivation of target RNA binding proteins (RBPs). In order to identify such candidate RBP targets, we screened a library of UAS overexpression lines [14] for inhibitors of dendrite pruning. In this screen, we identified *Drosophila* Mfap1, a spliceosome-associated protein. Control c4da neurons have long and branched dendrites at the third instar larval stage (Fig 1A) which are completely pruned at 18 h APF (Fig 1A’). Overexpression of Mfap1 did not cause major changes in the dendritic arbor at the third instar larval stage (Fig 1B and 1E). Strikingly, more than 70% of c4da neurons overexpressing Mfap1 still had dendrites attached to the cell body at 18 h APF (Fig 1B’, 1D and 1E). We also assessed the effects of Mfap1 knockdown with a previously validated RNAi construct [12]. Expression of this construct abrogated Mfap1 staining in c4da neurons (S1 Fig). Mfap1 knockdown did not cause dendritic changes at the third instar stage, and neurons expressing *mfap1* RNAi had also pruned all their dendrites at 18 h APF (Fig 1C,1C’, 1D and 1E).

### Mfap1 localizes to the nucleolus in neurons and accumulates upon VCP inhibition

**Fig 2:**
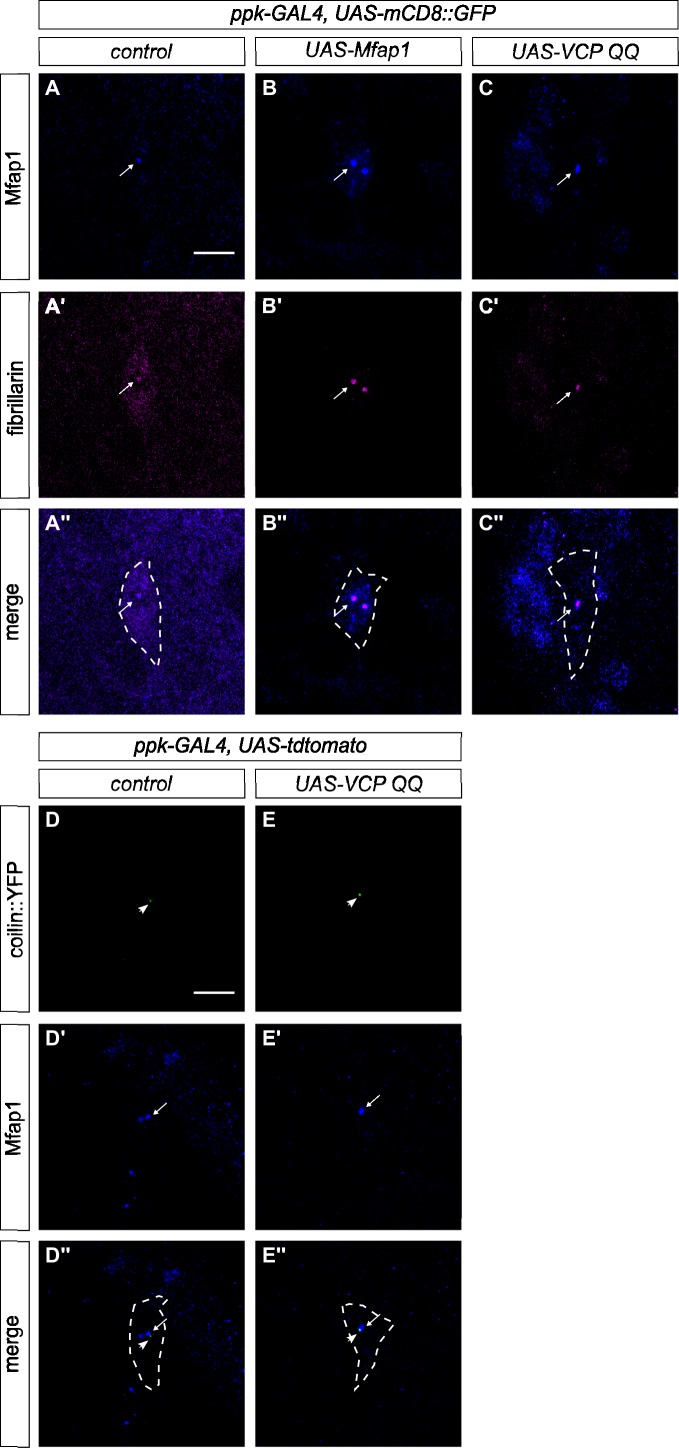
Mfap1 colocalizes with fibrillarin and is upregulated upon VCP inhibition. Mfap1 ((A)–(C)) or fibrillarin ((A’)–(C’)) were visualized by immunofluorescence in third instar larval c4da neurons. C4da neurons were visualized by expression of mCD8::GFP under *ppk-GAL4*. Panels (A”)–(C”) show merged Mfap1 and fibrillarin stainings, with the outline of the c4da neuron soma indicated by dashed lines. (A)–(A”) Control c4da neurons. (B)–(B”) C4da neurons overexpressing Mfap1.(C)–(C”) C4da neurons expressing dominant negative VCP QQ. (D)–(E”) The Cajal body marker coilin::YFP was expressed in c4da neurons under *ppk-GAL4*, and neuronal morphology was visualized by expression of tdtomato. Coilin::YFP ((D)–(E)) and Mfap1 ((D’)–(E’)) were visualized with anti-Mfap1 and anti-GFP antibodies, respectively. Panels (D”) and (E”) show merged coilin::YFP and Mfap1 stainings. Arrows indicate Mfap1 dots, arrowheads indicate coilin::YFP. The cell outline is indicated by dashed lines. (D)–(D”) Control c4da neurons. (E)–(E”) C4da neurons expressing VCP QQ. Scale bars are 10 μm.

We next asked if Mfap1 is expressed in c4da neurons. To this end, we performed immunofluorescence stainings with an Mfap1 antibody [12] on third instar larval filets. In control neurons, Mfap1 staining was detectable in a single small dot (and—rarely—also in two dots) in the nucleus (Fig 2A). Upon overexpression, Mfap1 was visible in one or two bright nuclear spots (Fig 2B). Several RNA-based processes take place in so-called nuclear bodies, dot-like structures in the nucleus, such as the Cajal body or the nucleolus. In order to assess whether the Mfap1 dots represented a previously characterized structure, we costained it with available markers for nuclear substructures. The nucleolus and Cajal body marker fibrillarin [15] could be detected in a single dot-like nuclear structure in control c4da neurons and always overlapped with Mfap1 (Fig 2A’ and 2A”). Interestingly the staining intensity of fibrillarin dots was also increased in neurons overexpressing Mfap1 (Fig 2B’ and 2B”).

In order to investigate whether Mfap1 was linked to VCP, we assessed Mfap1 expression in c4da neurons expressing dominant-negative VCP QQ (ATPase-dead VCP carrying mutations E335Q, E575Q) [9]. Under these conditions, Mfap1 dots were consistently brighter than in control neurons (Fig 2C and 2C”). Again, fibrillarin staining intensity followed that of Mfap1.

The Cajal body marker coilin-YFP [16] also localized to a small dot-like structure in the nucleus. These coilin::YFP dots were closely adjacent to Mfap1 dots, but did not overlap with them either in control neurons (Fig 2D and 2D”), or in neurons expressing VCP QQ (Fig 2E and 2E”). As both fibrillarin and coilin label the Cajal body, but only fibrillarin labels the nucleolus, our colocalization data suggest that Mfap1 localizes to the nucleolus. Moreover, our data suggest that Mfap1 levels might be controlled by VCP.

### Mfap1 and VCP can be found in a nucleotide-dependent complex

**Fig 3:**
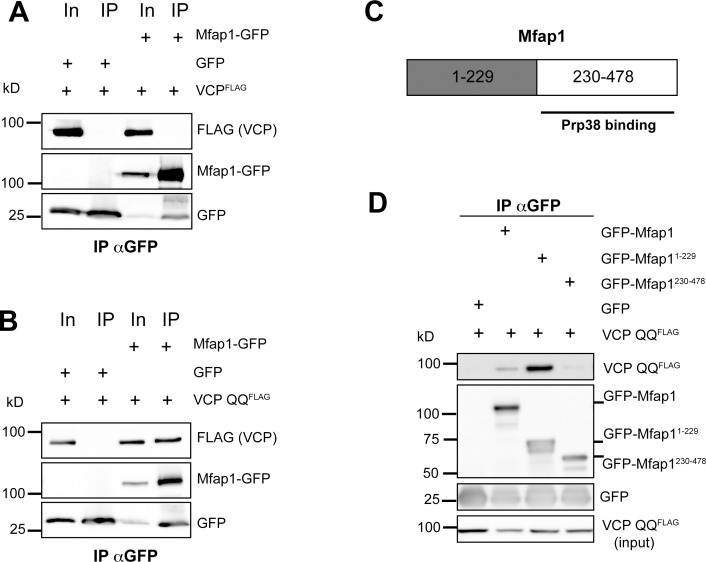
Mfap1 can form a complex with VCP. The indicated GFP-Mfap1 constructs (or GFP only as a control) were cotransfected with VCP^FLAG^ plasmids into S2 cells and immunoprecipitated with GFP antibodies. Precipitates were analysed by Western blotting with anti-GFP antibodies to detect GFP fusion proteins, and with anti-FLAG antibodies to detect coprecipitated VCP^FLAG^ constructs. (A) Mfap1-GFP IPs with wild type VCP. (B) Mfap1-GFP IPs with dominant-negative VCP QQ. (C) Schematic representation of the Mfap1 domain structure.(D) Immunoprecipitation of GFP-Mfap1^1-229^ or GFP-Mfap1^230-478^ coexpressed with VCP QQ. GFP bands in Mfap1-GFP samples are likely due to partial proteolysis after lysis.

As Mfap1 staining was enhanced by expression of the dominant-negative VCP QQ, we tested whether Mfap1 and VCP can form a complex. To this end, we co-transfected C-terminally GFP-tagged Mfap1 and FLAG-tagged VCP in S2 cells and performed co-immunoprecipitation experiments. We could not detect wild-type VCP in Mfap1-GFP immunopreciptates (Fig 3A), however, we could detect VCP in Mfap1-GFP immunoprecipitates when we coexpressed dominant-negative VCP QQ instead of wild type VCP (Fig 3B). Since VCP QQ, which is locked in the ATP-bound state, also acts as a substrate trap, these data suggest that Mfap1 binds to VCP either in a nucleotide-dependent manner, or very transiently.

We next wanted to identify the region of Mfap1 that mediates this interaction. Mfap1, a 478 amino acid protein, contains a conserved motif in its C-terminal ≈250 amino acids via which it binds to Prp38 [12]. The N-terminal part of 229 amino acids does not contain known motifs and appears to be largely unstructured [17]. We therefore repeated the co-immunoprecipitation experiments with GFP-tagged N- and a C-terminal fragments of Mfap1 that corresponded to amino acids 1–229 and 230–478, respectively. In these experiments, the smaller N-terminal fragment ran at a higher than expected molecular weight in SDS gels, a behavior sometimes seen with highly charged or unusually folded proteins (Fig 3D) (the calculated pI for Mfap1^1-229^ is 4.6). VCP QQ selectively and strongly bound to the N-terminal GFP-Mfap1^1-229^, but not the C-terminal GFP-Mfap1^230-478^ fragment (Fig 3D). Thus, the N-terminal part of Mfap1 mediates binding to VCP in the ATP-bound confirmation.

### Mfap1 interacts with TDP-43

**Fig 4:**
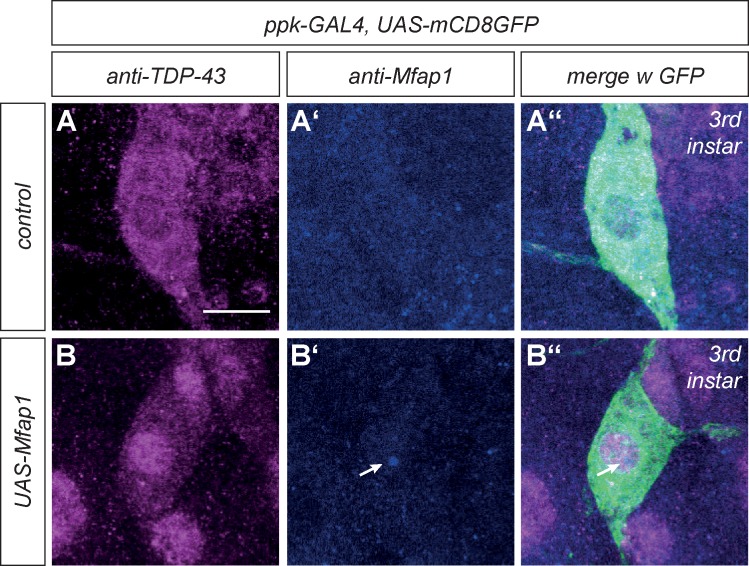
Mfap1 overexpression leads to TDP-43 relocalization to the nucleus. C4da neurons were visualized by mCD8::GFP expression under *ppk-GAL4*, and TDP-43 was visualized by immunofluorescence in control third instar larval c4da neurons ((A)–(A”)) or c4da neurons overexpressing Mfap1 ((B)–(B”)). Panels (A)–(B) show TDP-43 stainings, Panels (A’)–(B’) show Mfap1 stainings. Panels (A”)–(B”) show the merge with GFP.TDP-43 was not seen enriched in the nucleus of control c4da neurons (0/6), but in all c4da neurons overexpressing Mfap1 (5/5). Scale bar is 10 μm.

We previously showed that VCP inhibition in c4da neurons leads to mislocalization of the RNA-binding protein TDP-43 [8]. TDP-43 is a largely cytoplasmic protein in c4da neurons, but relocalizes to the nucleus upon inhibition of VCP or the 19S proteasome cap [8]. As Mfap1 interacts with VCP, we analyzed next whether Mfap1 manipulation also affects TDP-43. In control c4da neurons at the third instar larval stage, TDP-43 was localized diffusely in the cytoplasm (0/6 with nuclear staining) (Fig 4A and 4A”). In contrast, and similar to the situation upon VCP inhibition, TDP-43 was detected in the nucleus of c4da neurons upon Mfap1 overexpression (5/5 nuclear) (Fig 4B and 4B”).

**Fig 5:**
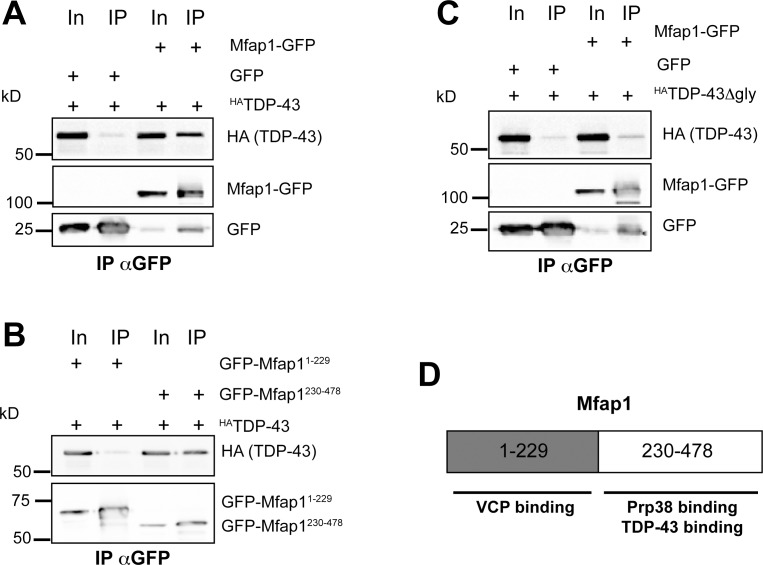
Mfap1 can be found in a complex with TDP-43. The indicated GFP-tagged Mfap1 constructs (or GFP only as a control) were cotransfected with HA-tagged TDP-43 plasmids into S2 cells and immunoprecipitated with antibodies against GFP. Precipitates were analysed by Western blotting with GFP antibodies to detect GFP fusion proteins, or HA antibodies to detect coprecipitated ^HA^TDP-43. (A) Coimmunoprecipitation of ^HA^TDP-43 with Mfap1-GFP.(B) Coimmunoprecipitations of ^HA^TDP-43 with GFP-Mfap1^1-229^ or GFP-Mfap1^230-478^. (C) Coimmunoprecipitation of ^HA^TDP-43 lacking the glycine-rich region (^HA^TDP-43 Δgly) with Mfap1-GFP. For better comparison, these lanes are from the same blot and exposure as the experiment in (A). (D) Schematic summary of Mfap1 domain interactions. GFP bands in Mfap1-GFP samples are likely due to partial proteolysis after lysis.

In order to follow up on this finding, we next tested whether Mfap1 could be found in a complex with TDP-43 in co-immunoprecipitation experiments from S2 cells co-transfected with tagged Mfap1 and TDP-43. HA-tagged TDP-43 could be detected in precipitates from cells expressing Mfap1-GFP, but not in control precipitates (Fig 5A). To identify the respective domains that mediate this interaction, we used deletion constructs of Mfap1 and TDP-43. When we used GFP-Mfap1^1-229^ and GFP-Mfap1^230-478^ in co-immunoprecipitation experiments with full length TDP-43, TDP-43 was precipitated with the C-terminal GFP-Mfap1^230-478^ encompassing the conserved motif, but not with N-terminal GFP-Mfap1^1-229^ that binds to VCP (Fig 5B and 5D). We also asked which region in TDP-43 mediates this interaction. TDP-43 contains two RNA-binding RRM domains and a so-called glycine-rich domain that has been implicated in protein-protein interactions within ribonucleoprotein particles [18]. When we deleted the glycine-rich region (amino acids 331–388, TDP-43Δgly) from *Drosophila* TDP-43, the interaction with Mfap1 was decreased almost to background levels, indicating that this domain also contributes to the interaction with Mfap1 (Fig 5C). When we used the N- and C-terminal GFP-Mfap1^1-229^ and GFP-Mfap1^230-478^ constructs in co-immunoprecipitation experiments with full length TDP-43, TDP-43 was precipitated with the C-terminal GFP-Mfap1^230-478^ encompassing the conserved motif, but not with N-terminal GFP-Mfap1^1-229^ that binds to VCP (Fig 5C). Thus, both the conserved C-terminal motif of Mfap1 and the glycine-rich region of TDP-43 are required for complex formation between the two factors.

### Mfap1 downregulation does not suppress the effects of VCP inhibition in c4da neurons

**Fig 6:**
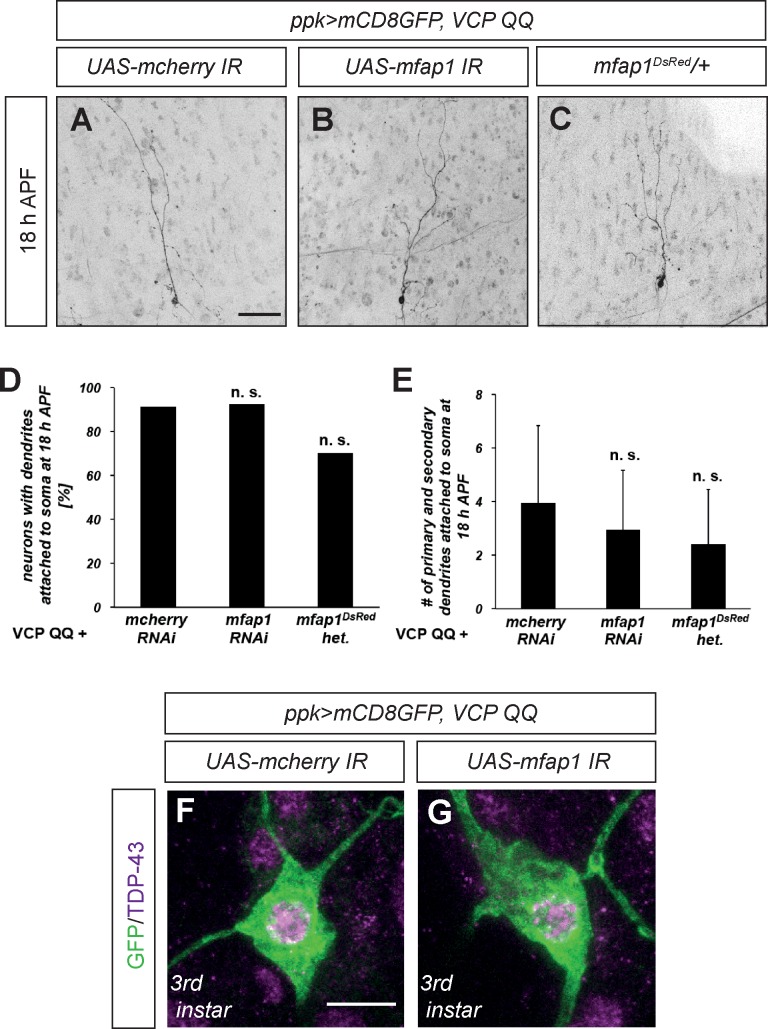
Mfap1 does not mediate VCP functions in c4da neurons. (A)—(E) Mfap1 is not required for c4da neuron dendrite pruning defects upon VCP inhibition. C4da neurons expressing CD8GFP, VCP QQ, and either *mcherry* RNAi as control or *mfap1* RNAi under *ppk-GAL4* were visualized at 18 h APF, and dendrite pruning defects were quantified.(A) C4da neuron coexpressing VCP QQ and *mcherry* RNAi at 18 h APF. (B) C4da neuron coexpressing VCP QQ and *mfap1* RNAi at 18 h APF. (C) C4da neuron expressing VCP QQ in a *mfap1*^*DsRed*^/+ background at 18 h APF. (D) Penetrance of dendrite pruning defects in experiments (A—C). Significance was assessed by Fisher’s exact test. (E) Number of primary and secondary dendrites still attached to the cell body in experiments (A—C). Significance was assessed by Wilcoxon’s test. (F), (G) Mfap1 does not mediate TDP-43 relocalization upon VCP inhibition. Third instar larval c4da neurons expressing mCD8::GFP, VCP QQ, and either *mcherry* RNAi as control or *mfap1* RNAi under *ppk-GAL4* were stained for TDP-43, and for GFP to visualize neuronal morphology. (F) Larval c4da neuron coexpressing VCP QQ and *mcherry* RNAi. (G) Larval c4da neuron coexpressing VCP QQ and *mfap1* RNAi. Scale bars are 50 μm in (A) and 10 μm in (F).

Our results so far have shown several phenotypic similarities between the loss of VCP function and a gain of Mfap1 function, as both manipulations cause c4da neuron dendrite pruning defects and a relocalization of TDP-43 from the cytoplasm to the nucleus. As Mfap1 also binds to VCP and TDP-43, we hypothesized that Mfap1 might be either a target for VCP in c4da neurons, or an adaptor that mediates binding between VCP and TDP-43. We reasoned that if VCP inhibition causes a gain of Mfap1 function which then leads to TDP-43 relocalization and/or dendrite pruning defects, then downregulation of Mfap1, either using RNAi-mediated knockdown or with a mutant, should suppress these effects. To test this hypothesis, we first assessed a potential genetic interaction between VCP and Mfap1 during c4da neuron dendrite pruning. We therefore co-expressed VCP QQ in c4da neurons together with *mcherry* RNAi as a control, or together with *mfap1* RNAi. As expected from previous work [8,9], co-expression of VCP QQ with the control RNAi caused strong dendrite pruning defects, with approximately 80% of neurons still having dendrites attached to the cell body at 18 h APF (Fig 6A, 6D and 6E). Coexpression of *mfap1* RNAi did not significantly alter the effect of VCP QQ, and a similar fraction of neurons still had dendrites attached to the cell body at 18 h APF (Fig 6B and 6D and 6E). Since in this experiment the RNAi effect might have been titrated somewhat by the presence of other UAS constructs, we also asked whether lowering *MFAP1* gene dosage could suppress the pruning defects caused by VCP inhibition. To this end, we used *mfap1*^*DsRed*^, a lethal P element insertion mutant in *mfap1* generated in our lab (see below). Since *mfap1*^*DsRed*^ is lethal prior to the pupal phase, we could only assess the effect of *mfap1*^*DsRed*^ heterozygousity. However, also this manipulation did not significantly suppress the pruning defects induced by VCP QQ (Fig 6C– 6E).

We next assessed the effect of Mfap1 knockdown on TDP-43 relocalization in third instar c4da neurons. As expected, TDP-43 localizes to the nucleus of c4da neurons upon expression of dominant-negative VCP QQ together with the *mcherry* control RNAi (Fig 6F). TDP-43 was also found in the nucleus when VCP QQ was coexpressed with *mfap1* RNAi (Fig 6G). These results indicate that Mfap1 does not mediate the effects of VCP in c4da neurons.

### Importance of Mfap1 domains

**Fig 7:**
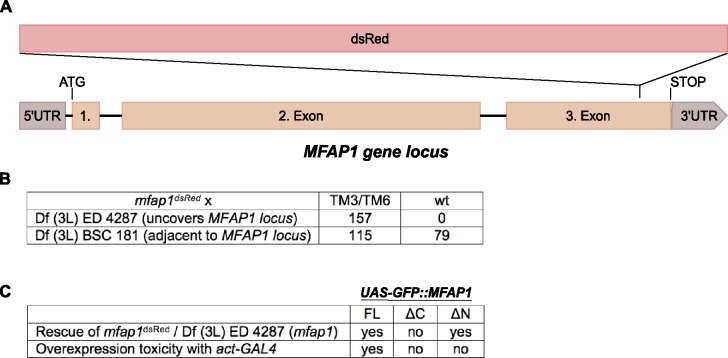
Identification of essential Mfap1 domains. (A) Schematic depicting the insertion site of the DsRed cassette in *mfap1*^*DsRed*^. (B) Table depicting the complementation analysis of *mfap1*^*DsRed*^ with *Df(3L)ED4287*, a deficiency uncovering *MFAP1*, and *Df(3L)BSC181*, which does not. *mfap1*^*DsRed*^ was crossed to the deficiency lines and the number of adult offspring carrying either the balancers, or the mutation *in trans* to the deficiency was counted. (C) Rescue of transheterozygous *mfap1*^*DsRed*^/*Df(3L)ED4287* flies by various Mfap1 constructs. Rescue constructs were provided from the indicated UAS-GFP-Mfap1 (full length or fragment) transgenes. “Rescue”indicates rescue to adult viability by either UAS expression of Mfap1 constructs under the control of *actin-GAL4*, or by leak expression from UAS insertions alone. “Overexpression toxicity”indicates no viable offspring carrying both *actin-GAL4* and UAS-GFP::Mfap1 (FL or fragments).

If Mfap1 does not mediate the effects of VCP in c4da neurons, what could be the significance of the observed Mfap1—VCP interactions? To address this question, we decided to assess genetically which domains of Mfap1 are critical for its function. We first aimed to generate a *mfap1* mutant. From an attempt to knock out the *MFAP1* locus using CRISPR/Cas9 and recombination-based insertion of a DsRed cassette [19], we recovered a line that had the DsRed cassette inserted in the last *MFAP1* coding exon. The cassette was inserted approximately 100 base pairs in front of the stop codon, thus disrupting the conserved C-terminal domain (Fig 7A). The *mfap1*^*DsRed*^ insertion was inviable over *Df(3L)ED4287*, a deficiency uncovering the *MFAP1* locus (Fig 7B), confirming that *MFAP1* is an essential gene, and the insertion either a null or a strong hypomorph. These data also support previous work showing that the interaction with Prp38 via its C-terminus is essential [12]. Based on our above biochemical analysis of Mfap1 domain interactions (summarized in Fig 5D), we then generated three GFP-tagged UAS-MFAP1 overexpression lines for rescue experiments: full-length Mfap1 (*UAS-GFP*::*Mfap1 FL*), the N-terminal fragment encompassing the VCP binding site (*UAS-GFP*::*Mfap1*^*1-229*^), and the conserved C-terminal fragment (*UAS-GFP*::*Mfap1*^*230-478*^). We expressed these under the control of the ubiquitous GAL4 driver *actin-GAL4* and asked which of them could rescue the lethality of transheterozygous *mfap1*^*DsRed*^/*Df(3L)ED4287* flies. At 25°C, the full-length Mfap1 construct rescued the lethality, but only in the absence of the GAL4 driver, i. e., through leak expression from the UAS promotor (Fig 7C). Flies carrying both *UAS-GFP*::*Mfap1 FL* and the GAL4 driver were not viable, indicating that overexpression of full length Mfap1 is toxic. Expression of the N-terminal UAS-GFP::Mfap1^1-229^ construct was not toxic, but did not rescue the lethality of transheterozygous mutants. The Mfap1 C-terminal fragment encompassing the conserved domain (*UAS-GFP*::*Mfap1*^*230-478*^) rescued the lethality and gave viable flies both in the absence and presence of *actin-GAL4* (Fig 7C). Thus, the conserved C-terminal domain of Mfap1 is necessary and sufficient for Mfap1 function. The N-terminal fragment of Mfap1 encompassing the VCP binding site is not essential for Mfap1 function, but confers overexpression toxicity in the context of full length Mfap1.

## Discussion

Here, we provide a characterization of Mfap1 expression in neurons, its interaction partners, and the functional importance of its domains. Using immunofluorescence, we found that Mfap1 localizes to a single dot-like structure in the nucleus of c4da neurons. Based on colocalization with fibrillarin, but not with coilin-YFP, our results indicate that Mfap1 localizes to the nucleolus, thus linking this structure to splicing in fly neurons. However, nuclear bodies in *Drosophila* neurons are not well characterized, and it will have to be confirmed whether they have the same marker distribution as in other tissues in the fly [16]. Interestingly, the levels of both Mfap1 and fibrillarin are sensitive to VCP manipulation and increase when VCP is inhibited. This, together with the biochemical data, suggests that Mfap1 might be a target of a VCP-dependent degradation pathway and could potentially implicate VCP in the control of splicing and/or nucleolus structure. However, this speculation will have to await functional confirmation.

In co-immunoprecipitation experiments with tagged proteins, we identified the chaperone VCP and the RNA-binding protein TDP-43 as interaction partners for Mfap1. While these interactions still need to be confirmed at the endogenous level, the fact that we could map them to discrete domains of Mfap1 suggests that they reflect real properties of Mfap1. Firstly, we found that Mfap1 can be recruited to a VCP-containing complex via a region in its N-terminal 229 amino acids. The interaction with VCP could only be detected with VCP QQ, which is locked in the ATP-bound confirmation. Nucleotide-dependence in VCP interactions has been described in some VCP adaptor proteins [20], albeit not as strong as observed here with Mfap1. Our results therefore probably reflect a transient substrate-like interaction of Mfap1 with VCP. The identified VCP binding region of Mfap1 is not required for viability but confers overexpression toxicity in the full length protein. Thus, this domain–and possibly the VCP interaction—might in some way act to make Mfap1 more efficient or active. This could occur through VCP-mediated stabilization of Mfap1, which would be consistent with the observation that locking the Mfap1-VCP interaction in c4da neurons increases Mfap1 levels (Fig 2). The observation that the C-terminal domain of Mfap1 can rescue the mutant, but is not toxic upon overexpression (Fig 7), could indicate that this domain is more short-lived than the full length Mfap1 containing the N-terminal VCP interaction site.

The conserved C-terminal region of Mfap1 is essential for Mfap1 function. This region was previously shown to bind to Prp38 [12], another essential splicing factor, indicating that recruitment of Prp38 might be the essential function of Mfap1. We also identified TDP-43 as a new interactor for the Mfap1 C-terminus. Thus, the Mfap1 C-terminus might act as a platform for multiple splicing factors, such as accessory alternative splicing regulators. It will be interesting to see if Mfap1 can bind to both Prp38 and TDP-43 simultaneously. Taken together, our data suggest new entry points into splicing research.

## Materials and methods

### Fly stocks

Flies were kept at 25°C for all experiments. Fly stocks were UAS-Mfap1 (FlyORF F001460), UAS-Mfap1 RNAi (VDRC 15610), UAS-coilin::YFP [16], *ppk-GAL4* [21], UAS-tdtomato [22], UAS-VCP QQ [9], UAS-mcherry RNAi (Bloomington 35785).

### Generation of the *mfap1*^*DsRed*^ mutant

gRNAs corresponding to sites in the 5’UTR and 100 bp in front of the *MFAP1* stop codon were designed with the CRISPR Optimal Target Finder (http://tools.flycrispr.molbio.wisc.edu/targetFinder/index.php) and cloned into pCFD3. Forward gRNA sequences were:

gRNA1: gtcgAAAGATTTGCTTATTACCCG,

gRNA2: gtcgCTGGATGACTCGGCGTACCA.

Mfap1 homology arms (ca. 1.5 kb outside of the respective 5’ and 3’ gRNAs) were cloned into pBluescript such that they flanked the 3P3-DsRed marker. Homology arm primers were:

left arm, CGTCTCAggacGTTTAATTGCTGGTTCTTGG (fw), CGTCTCActggGTAATAAGCAAATCTTTGTATTTATTCC (rev);

right arm, CGTCTCAtgttTACGCCGAGTCATCCAGCAA (fw), CGTCTCAgcatAGGCAACTGTGGAAAAGCCT (rev).

The gRNA plasmids and the homology cassette were injected into *act-Cas9*, *lig4*^*-/-*^ flies [19]. Injectants were crossed to a third chromosome balancer and offspring was screened for red fluorescent eyes. Two candidates were then assessed for lethality over *Df(3L)ED4287* (Bloomington 8096) uncovering the *MFAP1* locus, and characterized by PCR and sequencing. A line, designated *mfap1*^*DsRed*^, was recovered that had the cassette inserted at the 3’ CRISPR target approximately 100 base pairs in front of the *MFAP1* stop codon. Primers for genomic PCR were:

upstream PCR: TCCACGAATGGCCGGAAAAT and XZ85 [17],

downstream PCR: XZ120 [17], CTACAGGAGCACACGCTCAA.

### Cloning and transgenes

Sequences corresponding to full length Mfap1 (1–478), Mfap1^1-229^ and Mfap1^230-478^ were cloned into pUAST attB GFP and inserted into the second chromosome 44F landing site [23]. pUAST attB ^HA^TDP-43 Δ331–388 (Δgly) was generated by PCR from pUAST attB ^HA^TDP-43 [8].

### Microscopy

Live images of larval or pupal c4da neurons at 18 h APF were taken on a Zeiss LSM710 confocal microscope, and dendrite and pruning phenotypes were quantified as previously described [9].

### Antibodies and immunohistochemistry

Antibody stainings of larval filets were done as decribed [8] except that filets were fixed with Bouin’s solution for three minutes for Mfap1 stainings. Antibodies were guinea pig anti-Mfap1 [12] 1:200, mouse anti-Fibrillarin 72B9 [24] 1:5, rabbit anti-dTDP-43 [25] 1:200, rabbit anti-GFP (Invitrogen) 1:1000, rabbit anti-DsRed (Clontech) 1:1000. Images were taken on an LSM710 microscope.

### Immunoprecipitation

The indicated UAS plasmids were cotransfected with actC5-GAL4 into S2 cells and allowed to express for 72 hours. Lysis and immunoprecipitation were performed according to standard methods [9]. For immunoprecipitations, rabbit anti-GFP antibodies (Invitrogen) were used (1 μg), Western blots were probed with rabbit anti-GFP JL8 (Clontech) 1:1000, mouse anti-FLAG M2 (Sigma) 1:5000, and mouse anti-HA (BioLegend) 1:1000. N- or C-terminally GFP-tagged Mfap1 versions were used interchangeably.

## Supporting information

S1 Fig Validation of Mfap1 RNAi. Control c4da neurons (A, A’) or c4da neurons expressing Mfap1 RNAi under *ppk-GAL4* (B, B’) were stained for Mfap1 at the third instar larval stage. Panels (A), (B) show Mfap1 stainings, panels (A’), (B’) show the merge with GFP to depict the c4da neuron soma. The soma is also indicated by a dashed line in panels (A) and (B). The arrow in (A) depicts the characteristic Mfap1 dot.
(EPS)

S2 Fig Original uncropped blots for Mfap1-VCP immunoprecipitations (Fig 3A and 3B). Cotransfected UAS constructs are indicated on top. A FLAG blot for FLAG-tagged VCP versions. Lanes 1–4 were shown in Fig 3A, lanes 5–8 in Fig 3B. A’ GFP blot for Mfap1-GFP or GFP as control.
(EPS)

S3 Fig Original uncropped blots for GFP-Mfap1 fragment immunoprecipitation (Fig 3C). Cotransfected UAS constructs are indicated on top. A FLAG blot for FLAG-tagged VCP QQ. Lanes 1–4 represent input samples shown in Fig 3C, lanes 6–9 IP samples. A’ GFP blot for Mfap1-GFP constructs.
(EPS)

S4 Fig Original uncropped blots for Mfap1-TDP-43 immunoprecipitations (Fig 5A and 5B). Cotransfected UAS constructs are indicated on top. A HA blot for HA-tagged TDP-43 versions. Lanes 1–4 were shown in Fig 5A, lanes 5–8 in Fig 5B. A’ GFP blot for Mfap1-GFP or GFP as control.
(EPS)

S5 Fig Original uncropped blots for GFP-Mfap1 fragment coimmunoprecipitations with TDP-43 (Fig 5C). Cotransfected UAS constructs are indicated on top. A HA blot for HA-tagged TDP-43 versions. Lanes 5–8 are shown in Fig 5C. A’ GFP blot for GFP-Mfap1 fragments or GFP as control.
(EPS)

S6 Fig PCR verification of *mfap1*^*DsRed*^ mutant. PCRs were done on genomic DNA from control flies (*Actin-Cas9*) or *mfap1*^*DsRed*^/Tm6b mutant flies. A Genomic PCR using primers for the Mfap1 upstream region and the DsRed cassette. The expected 3.2 kb band is only seen in the mutant. B Genomic PCR using primers for the Mfap1 downstream region and the DsRed cassette. The expected 0.9 kb band is only seen in the mutant.
(EPS)
